# Genomic Sequencing of Ranaviruses Isolated from Edible Frogs (*Pelophylax esculentus*)

**DOI:** 10.1128/genomeA.01015-17

**Published:** 2017-09-21

**Authors:** Ellen Ariel, Kuttichantran Subramaniam, Kamonchai Imnoi, Preeyanan Sriwanayos, M. Shamim Ahasan, Niels J. Olesen, Manfrin Amedeo, Anna Toffan, Thomas B. Waltzek

**Affiliations:** a College of Public Health, Medical and Veterinary Sciences, James Cook University, Townsville, Queensland, Australia; b Department of Infectious Diseases and Immunology, College of Veterinary Medicine, University of Florida, Gainesville, Florida, USA; c Technical University of Denmark, National Veterinary Institute, Kgs. Lyngby, Denmark; d National Reference Laboratory for Fish Diseases, Istituto Zooprofilattico Sperimentale delle Venezie, Legnaro, Italy

## Abstract

Ranaviruses were isolated from wild edible frogs (*Pelophylax esculentus*) during epizootics in Denmark and Italy. Phylogenomic analyses revealed that these isolates are closely related and belong to a clade of ranaviruses that includes the *Andrias davidianus* ranavirus (ADRV), common midwife toad ranavirus (CMTV), *Testudo hermanni* ranavirus (THRV), and pike-perch iridovirus (PPIV).

## GENOME ANNOUNCEMENT

The earliest reported occurrence of ranaviral disease in amphibians in Europe resulted in recurrent, low-level mortality in wild-caught edible frogs in a commercial operation in Croatia from 1970 to 1981 (1). Other ranaviral isolates from edible frogs have been obtained from tadpoles collected from the wild during disease outbreaks in Italy in 2002 (REV 282/I02) and from free-living adults collected in Denmark in 2008 (PEV_DK1) (2, 3). Mortality events in *Rana temporaria* in the United Kingdom in the 1990s (4) and, more recently, in common midwife toads (*Alytes obstetricans*) in Spain (5) and *Pelophylax* spp. in The Netherlands (6) have been added to the list of amphibian ranaviral outbreaks in Europe.

The two isolates PEV_DK1 and REV 282/I02 were ampliﬁed in epithelioma papulosum cyprini cells until the cytopathic effect was complete, and then the resulting supernatant was clarified and the total nucleic acid was purified from the clarified supernatant, as previously described (7). DNA libraries were prepared using the Nextera XT DNA kit (Illumina), and sequencing was performed using a v3 chemistry 600-cycle kit on an Illumina MiSeq platform. *De novo* assembly of the paired-end reads in SPAdes (8) produced contiguous consensus sequences of 107,392 bp with G+C content of 55.31% and 107,444 bp with G+C content of 56.03% for PEV_DK1 and REV 282/I02, respectively.

The genomes of the two isolates were annotated using Genome Annotation Transfer Utility (GATU) (9) with *Frog virus 3* (GenBank accession no. NC_005946) as the reference genome. Additional putative open reading frames (ORFs) were identified using GenemarkS (10), and gene functions were predicted based on BLASTP searches against the NCBI GenBank non-redundant protein sequence database. A total of 99 putative ORFs were predicted in PEV_DK1 and 101 in REV 282/I02 compared to 101 ORFs in *Andrias davidianus* ranavirus (ADRV) (Genbank accession no. KC865735), 104 ORFs in common midwife toad ranavirus (CMTV) (GenBank accession no. JQ231222 and KP056312), 75 in *Testudo hermanni* ranavirus (THRV) (GenBank accession no. KP266741), and 109 in pike-perch iridovirus (PPIV) (GenBank accession no. KX574341). Comparative genomic analyses revealed that these two ranaviruses are closely related, except for a nonsense mutation in a gene encoding a hypothetical protein (orthologous to ORF51 in REV 282/I02) and the absence of a gene encoding a hypothetical protein (orthologous to ORF95 in REV 282/I02) in PEV_DK1. An analysis of locally collinear blocks (LCB) in Mauve (11) revealed that the genomes of PEV_DK1 and REV 282/I02 display the same genome arrangement as ADRV, CMTV, THRV, and PPIV (12). Maximum likelihood phylogenetic analyses based on the concatenated genome-wide LCB alignments revealed that the Italian and Danish ranaviral isolates from edible frogs belong to a clade of ranaviruses that includes ADRV, CMTV, THRV, and PPIV.

The repeated isolation, separated by time and space, of nearly identical ranaviral strains from edible frogs indicates that this species may serve as an important natural host. The detection of closely related strains in Chinese giant salamander (*Andrias davidianus*), common midwife toad (*Alytes obstetricans*), Hermann’s tortoise (*Testudo hermanni*), and pike-perch (*Sander lucioperca*) underscores the low host specificity of these ranaviruses.

### Accession number(s).

The complete genome sequences of PEV_DK1 and REV 282/I02 have been deposited in GenBank under the accession numbers MF538627 and MF538628, respectively.
